# Crystal structure of tris­[μ_2_-bis­(di­phenyl­phosphan­yl)methane-κ^2^ *P*:*P*′]di-μ_3_-iodido-tris­ilver(I) iodide–*N*-phenyl­thio­urea (1/1)

**DOI:** 10.1107/S2056989015017120

**Published:** 2015-10-03

**Authors:** Yupa Wattanakanjana, Arunpatcha Nimthong-Roldán, Suthida Palavat, Walailak Puetpaiboon

**Affiliations:** a Department of Chemistry, Faculty of Science, Prince of Songkla University, Hat Yai, Songkhla 90112, Thailand; b Department of Chemistry, Youngstown State University, 1 University Plaza, 44555, Youngstown, OH, USA

**Keywords:** crystal structure, *N*,*N*′-phenyl­thio­urea, silver complex, hydrogen bonding

## Abstract

The title complex, [Ag_3_I_2_(C_25_H_22_P_2_)_3_]I·C_7_H_8_N_2_S, comprises a trinuclear [Ag_3_I_2_(C_25_H_22_P_2_)_3_]^+^ unit, an I^−^ anion and one *N*,*N*′-phenyl­thio­urea mol­ecule (ptu). Two μ_3_-bridging I^−^ anions are linked by three Ag^I^ ions, leading to the formation of a dicapped triangular motif with Ag⋯Ag separations in the range 3.0823 (5)–3.2999 (5) Å. Each Ag^I^ atom exhibits a distorted tetra­hedral geometry, with coordination to two I atoms and two P atoms from bis­(di­phenyl­phosphan­yl)methane ligands. In the crystal, the I^−^ anion is linked to the ptu mol­ecule through two N—H⋯I hydrogen bonds [graph-set motif *R*_2_^1^(6)]. These N—H⋯I hydrogen bonds, in addition to weak C—H⋯S and C—H⋯I hydrogen bonds, form zigzag chains along [010]. Two of the phenyl rings of two dppm ligands are disordered over two sets of sites with refined occupancies of 0.557 (16) and 0.443 (16).

## Related literature   

For bis­(di­phenyl­phosphino)methane (dppm) complexes formed with metal(I) ions, see: Bera *et al.* (1998 ▸); Matsumoto *et al.* (2001 ▸); Nicola *et al.* (2005 ▸). For the complex [Ag_3_(C_25_H_22_P_2_)_3_(μ_3_-Br)_2_]^+^, see: Nimthong-Roldán *et al.* (2015 ▸).
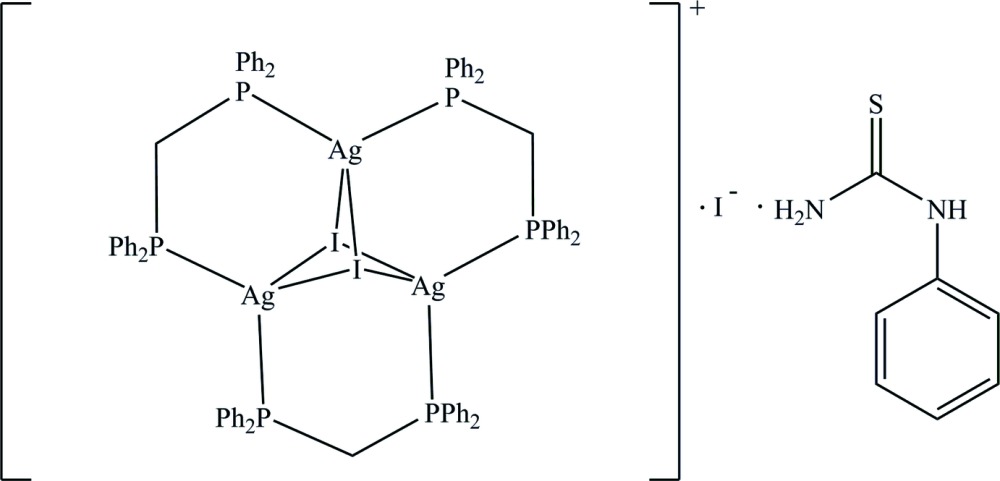


## Experimental   

### Crystal data  

[Ag_3_I_2_(C_25_H_22_P_2_)_3_]I·C_7_H_8_N_2_S
*M* *_r_* = 2009.62
Monoclinic, 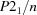

*a* = 10.8177 (4) Å
*b* = 28.5680 (11) Å
*c* = 26.1639 (11) Å
β = 95.315 (2)°
*V* = 8050.9 (5) Å^3^
*Z* = 4
Cu *K*α radiation
μ = 16.53 mm^−1^
*T* = 296 K
0.14 × 0.04 × 0.03 mm

### Data collection  

Bruker Prospector CCD diffractometer
Absorption correction: multi-scan (*SADABS*; Bruker, 2013 ▸) *T*_min_ = 0.394, *T*_max_ = 0.753
82145 measured reflections
14160 independent reflections
12277 reflections with *I* > 2σ(*I*)
*R*_int_ = 0.056

### Refinement  

*R*[*F* ^2^ > 2σ(*F* ^2^)] = 0.039
*wR*(*F* ^2^) = 0.104
*S* = 1.08
14160 reflections
983 parameters
216 restraints
H-atom parameters constrained
Δρ_max_ = 1.16 e Å^−3^
Δρ_min_ = −1.04 e Å^−3^

### 

Data collection: *APEX2* (Bruker, 2013 ▸); cell refinement: *SAINT* (Bruker, 2013 ▸); data reduction: *SAINT*; program(s) used to solve structure: *SHELXS97* (Sheldrick, 2008 ▸); program(s) used to refine structure: *SHELXL2014* (Sheldrick, 2015 ▸) and *SHELXLE* (Hübschle *et al.*, 2011 ▸); molecular graphics: *Mercury* (Macrae *et al.*, 2008 ▸); software used to prepare material for publication: *publCIF* (Westrip, 2010 ▸).

## Supplementary Material

Crystal structure: contains datablock(s) I. DOI: 10.1107/S2056989015017120/lh5785sup1.cif

Structure factors: contains datablock(s) I. DOI: 10.1107/S2056989015017120/lh5785Isup2.hkl

**Fig. 1.**
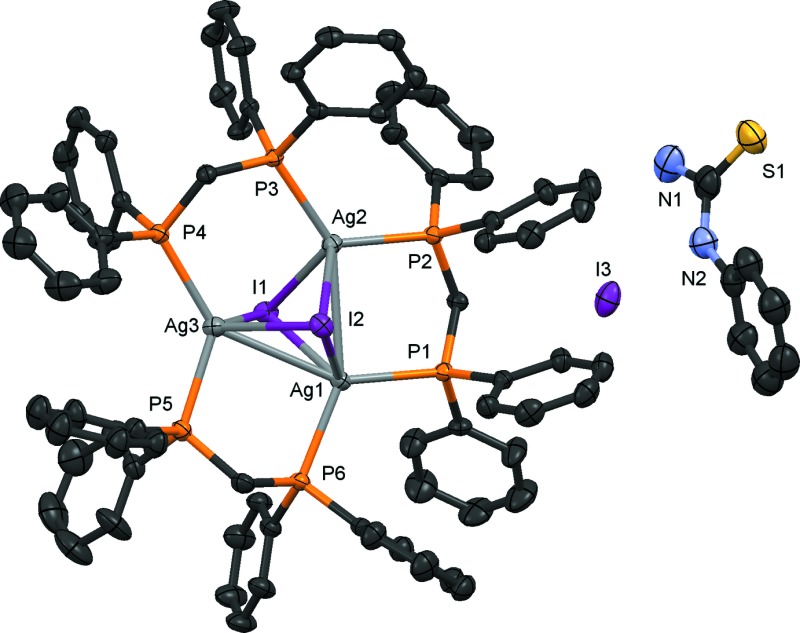
The molecular structure with displacement ellipsoids drawn at the 50% probability level. All H atoms and the minor component of disorder are omitted for clarity.

. DOI: 10.1107/S2056989015017120/lh5785fig1.tif
The mol­ecular structure with displacement ellipsoids drawn at the 50% probability level. All H atoms and the minor component of disorder are omitted for clarity.

**Fig. 2.**
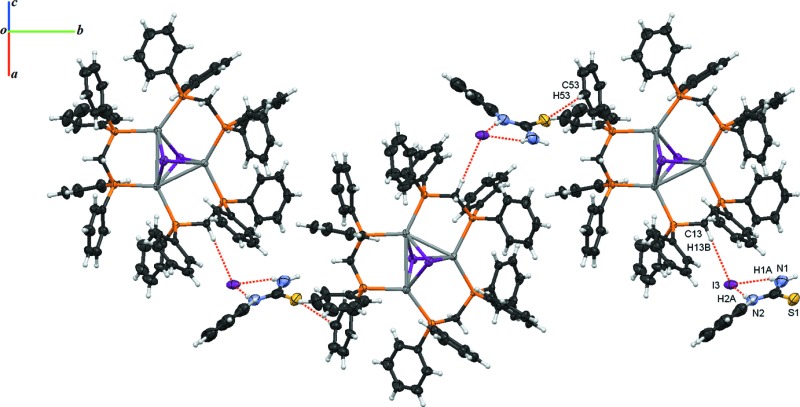
Part of the crystal structure showing intermolecular N—H···I, C—H···S and C—H···I hydrogen bonds as dashed lines, forming a chain along [010].

. DOI: 10.1107/S2056989015017120/lh5785fig2.tif
Part of the crystal structure showing inter­molecular N—H⋯I, C—H⋯S and C—H⋯I hydrogen bonds as dashed lines, forming a chain along [010].

CCDC reference: 1424053

Additional supporting information:  crystallographic information; 3D view; checkCIF report

## Figures and Tables

**Table 1:** Hydrogen-bond geometry (, )

*D*H*A*	*D*H	H*A*	*D* *A*	*D*H*A*
N1H1*A*I3	0.86	2.82	3.633(9)	159
N2H2*A*I3	0.86	2.72	3.568(6)	170
C53H53S1^i^	0.99	2.88	3.67(2)	143
C13H13*B*I3	0.99	3.04	3.933(4)	153

Symmetry code: (i) 
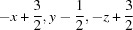
.

## supplementary crystallographic information

### S1. Comment

Complexes of bis­(di­phenyl­phosphino)methane (dppm) with metal(I) ions have been extensively studied (Bera *et al.* 1998; Matsumoto *et al.* 2001; Nicola *et al.* 2005). Recently, we reported the complex which we prepared by reacting silver (I) bromide and dppm, followed by the addition of *N*,*N*'-phenyl­thio­urea (ptu) in aceto­nitrile solvent. An unexpected complex [Ag_3_(C_25_H_22_P_2_)_3_(µ_3_-Br)_2_]^+^ unit was formed with uncoordinated ptu (Nimthong-Roldán *et al.* 2015). Herein, we present the complex formed using silver(I) iodide instead of silver(I) bromide under the same conditions.

The title complex consists of a trinuclear [Ag_3_(C_25_H_22_P_2_)_3_(µ_3_-I)_2_]^+^ unit, a discrete I^-^ anion and one *N*,*N*′-phenyl­thio­urea molecule (ptu). Three Ag^I^ ions in the corners of the trianglular plane are linked via two bridging µ_3_-I anions, forming a dicapped triangular geometry with a Ag···Ag separations range of 3.0823 (5)–3.2999 (5) Å. Each Ag^I^ ion coordinates to two iodide ions and two P atoms of two dppm ligands in a distorted tetra­hedral geometry (Fig 1). In the crystal, two N—H···I hydrogen bonds exist between the iodide anion and ptu molecule [graph-set motif R^2^_1_(6)] (Table 1). These hydrogen bonds are in turn connected via weak C—H···S and C—H···I hydrogen bonds, forming a zig-zag chain along [010] (Fig. 2, Table 1). Two of the phenyl rings from two dppm ligands are disordered over two sites with refined occupancies 0.557 (16) and 0.443 (16).

### S2. Synthesis and crystallization

Bis(di­phenyl­phosphino)methane, dppm, (0.1 g, 0.26 mmol) was dissolved in 30 ml of aceto­nitrile at 343 K and then silver(I) iodide, AgI, (0.06 g, 0.26 mmol) was added. The mixture was stirred for 4 hr and then *N*,*N*′-phenyl­thio­urea, ptu, (0.04 g, 0.26 mmol) was added and the new reaction mixture was heated under reflux for 6 hr during which the precipitate gradually disappeared. The resulting clear solution was filtered and left to evaporate at room temperature. The crystalline complex, which deposited upon standing for several days, was filtered off and dried in vacuo.

### S3. Refinement details

H atoms bonded to C and N atoms were included in calculated positions and were refined with a riding model using distances of 0.95 Å (aryl H), and *U*_iso_(H) = 1.2*U*_eq_(C); 0.99 Å (CH_2_) and *U*_iso_(H) = 1.5*U*_eq_(C); 0.88 Å (NH), and *U*_iso_(H) = 1.2*U*_eq_(N). Two phenyl rings from two dppm ligands are disordered. The geometry of the minor component of each pair of disordered phenyl rings was restrained to be similar to that of the major componnet (within a standard deviation of 0.02 Å). Carbon atoms of one phenyl ring were restrained with effective standard deviation 0.01 to have the same Uij components. To ensure satisfactory refinement the atoms of each disorder component of the phenyl rings were restrained to lie within a common plane. The overall ratio of the two components of disorder, refined with the same free variable, is 0.557 (16):0.443 (16).

### Crystal data

**Table:**

[Ag_3_I_2_(C_25_H_22_P_2_)_3_]I·C_7_H_8_N_2_S	*F*(000) = 3944
*M**_r_* = 2009.62	*D*_x_ = 1.658 Mg m^−^^3^
Monoclinic, *P*2_1_/*n*	Cu *K*α radiation, λ = 1.54178 Å
*a* = 10.8177 (4) Å	Cell parameters from 9031 reflections
*b* = 28.5680 (11) Å	θ = 2.3–66.5°
*c* = 26.1639 (11) Å	µ = 16.53 mm^−^^1^
β = 95.315 (2)°	*T* = 296 K
*V* = 8050.9 (5) Å^3^	Block, colourless
*Z* = 4	0.14 × 0.04 × 0.03 mm

### Data collection

**Table:**

Bruker Prospector CCD diffractometer	14160 independent reflections
Radiation source: I-mu-S microsource X-ray tube	12277 reflections with *I* > 2σ(*I*)
Laterally graded multilayer (Goebel) mirror monochromator	*R*_int_ = 0.056
ω and φ scans	θ_max_ = 67.2°, θ_min_ = 2.3°
Absorption correction: multi-scan (*SADABS*; Bruker, 2013)	*h* = −12→12
*T*_min_ = 0.394, *T*_max_ = 0.753	*k* = −34→33
82145 measured reflections	*l* = −30→31

### Refinement

**Table:**

Refinement on *F*^2^	Primary atom site location: structure-invariant direct methods
Least-squares matrix: full	Secondary atom site location: difference Fourier map
*R*[*F*^2^ > 2σ(*F*^2^)] = 0.039	Hydrogen site location: inferred from neighbouring sites
*wR*(*F*^2^) = 0.104	H-atom parameters constrained
*S* = 1.08	*w* = 1/[σ^2^(*F*_o_^2^) + (0.0506*P*)^2^ + 12.1838*P*] where *P* = (*F*_o_^2^ + 2*F*_c_^2^)/3
14160 reflections	(Δ/σ)_max_ = 0.002
983 parameters	Δρ_max_ = 1.16 e Å^−^^3^
216 restraints	Δρ_min_ = −1.04 e Å^−^^3^

### Special details

**Table:**

Geometry. All e.s.d.'s (except the e.s.d. in the dihedral angle between two l.s. planes) are estimated using the full covariance matrix. The cell e.s.d.'s are taken into account individually in the estimation of e.s.d.'s in distances, angles and torsion angles; correlations between e.s.d.'s in cell parameters are only used when they are defined by crystal symmetry. An approximate (isotropic) treatment of cell e.s.d.'s is used for estimating e.s.d.'s involving l.s. planes.

### Fractional atomic coordinates and isotropic or equivalent isotropic displacement parameters (Å^2^)

**Table:**

	*x*	*y*	*z*	*U*_iso_*/*U*_eq_	Occ. (<1)
I1	0.09573 (3)	0.07662 (2)	0.74036 (2)	0.04248 (9)	
I2	0.49507 (3)	0.04371 (2)	0.81433 (2)	0.04443 (9)	
I3	0.39502 (4)	0.18325 (2)	0.50190 (2)	0.07625 (14)	
Ag1	0.32400 (3)	0.03008 (2)	0.71995 (2)	0.04176 (9)	
Ag2	0.33079 (3)	0.12525 (2)	0.77564 (2)	0.03938 (9)	
Ag3	0.23020 (4)	0.03137 (2)	0.83250 (2)	0.04526 (10)	
S1	0.8130 (3)	0.30895 (9)	0.55157 (10)	0.1102 (8)	
N1	0.5783 (8)	0.2843 (3)	0.5464 (3)	0.106 (2)	
H1A	0.5193	0.2643	0.5407	0.127*	
H1B	0.5620	0.3126	0.5550	0.127*	
N2	0.7041 (6)	0.2262 (2)	0.5281 (2)	0.0783 (16)	
H2A	0.6339	0.2121	0.5223	0.094*	
P1	0.39878 (11)	0.06755 (4)	0.64386 (4)	0.0340 (2)	
C1	0.3240 (5)	0.04256 (16)	0.58416 (18)	0.0409 (11)	
C2	0.2052 (6)	0.0260 (3)	0.5856 (2)	0.0676 (17)	
H2	0.1655	0.0292	0.6154	0.081*	
C3	0.1443 (7)	0.0045 (3)	0.5432 (3)	0.088 (2)	
H3	0.0644	−0.0071	0.5447	0.106*	
C4	0.2018 (8)	0.0004 (3)	0.4990 (3)	0.086 (2)	
H4	0.1605	−0.0134	0.4701	0.103*	
C5	0.3164 (8)	0.0163 (4)	0.4975 (3)	0.099 (3)	
H5	0.3560	0.0126	0.4677	0.119*	
C6	0.3785 (7)	0.0384 (3)	0.5396 (2)	0.080 (2)	
H6	0.4577	0.0504	0.5374	0.096*	
C7	0.5644 (4)	0.06579 (16)	0.63783 (17)	0.0374 (10)	
C8	0.6341 (5)	0.03319 (19)	0.6672 (2)	0.0512 (12)	
H8	0.5946	0.0128	0.6881	0.061*	
C9	0.7616 (6)	0.0304 (2)	0.6660 (3)	0.0658 (16)	
H9	0.8076	0.0086	0.6861	0.079*	
C10	0.8188 (6)	0.0600 (3)	0.6348 (2)	0.0640 (16)	
H10	0.9044	0.0583	0.6339	0.077*	
C11	0.7528 (5)	0.0922 (2)	0.6048 (2)	0.0593 (15)	
H11	0.7934	0.1119	0.5835	0.071*	
C12	0.6258 (5)	0.0954 (2)	0.6062 (2)	0.0484 (12)	
H12	0.5809	0.1175	0.5859	0.058*	
C13	0.3510 (5)	0.12916 (15)	0.63727 (17)	0.0366 (10)	
H13A	0.2622	0.1304	0.6277	0.044*	
H13B	0.3920	0.1432	0.6096	0.044*	
P2	0.38689 (11)	0.16385 (4)	0.69593 (4)	0.0341 (2)	
C14	0.5505 (5)	0.17912 (16)	0.69543 (17)	0.0401 (10)	
C15	0.6363 (5)	0.15182 (19)	0.72515 (19)	0.0478 (12)	
H15	0.6090	0.1283	0.7459	0.057*	
C16	0.7618 (6)	0.1597 (2)	0.7238 (2)	0.0628 (15)	
H16	0.8187	0.1406	0.7428	0.075*	
C17	0.8037 (6)	0.1952 (3)	0.6947 (2)	0.0705 (19)	
H17	0.8884	0.2005	0.6941	0.085*	
C18	0.7194 (7)	0.2231 (3)	0.6665 (3)	0.0731 (19)	
H18	0.7474	0.2476	0.6472	0.088*	
C19	0.5942 (6)	0.2152 (2)	0.6665 (2)	0.0564 (14)	
H19	0.5382	0.2342	0.6470	0.068*	
C20	0.2963 (5)	0.21695 (16)	0.68020 (19)	0.0426 (11)	
C21	0.2735 (7)	0.2341 (2)	0.6309 (2)	0.0631 (16)	
H21	0.3122	0.2203	0.6045	0.076*	
C22	0.1944 (8)	0.2714 (2)	0.6202 (3)	0.077 (2)	
H22	0.1810	0.2828	0.5869	0.093*	
C23	0.1348 (7)	0.2918 (2)	0.6593 (3)	0.078 (2)	
H23	0.0799	0.3165	0.6521	0.093*	
C24	0.1568 (7)	0.2758 (2)	0.7079 (3)	0.0747 (19)	
H24	0.1167	0.2895	0.7340	0.090*	
C25	0.2398 (6)	0.23871 (19)	0.7191 (2)	0.0596 (15)	
H25	0.2568	0.2287	0.7529	0.072*	
P3	0.31631 (10)	0.16684 (4)	0.85759 (4)	0.0306 (2)	
C26	0.4537 (4)	0.20236 (15)	0.87508 (17)	0.0341 (9)	
C27	0.5244 (4)	0.21633 (17)	0.83608 (19)	0.0413 (10)	
H27	0.5020	0.2067	0.8025	0.050*	
C28	0.6280 (5)	0.24443 (19)	0.8466 (2)	0.0520 (13)	
H28	0.6734	0.2540	0.8200	0.062*	
C29	0.6640 (5)	0.25822 (18)	0.8956 (2)	0.0529 (13)	
H29	0.7344	0.2767	0.9024	0.063*	
C30	0.5955 (5)	0.24460 (19)	0.9349 (2)	0.0521 (13)	
H30	0.6198	0.2542	0.9683	0.063*	
C31	0.4909 (5)	0.21680 (18)	0.9253 (2)	0.0451 (11)	
H31	0.4456	0.2078	0.9521	0.054*	
C32	0.1869 (4)	0.20864 (15)	0.85560 (16)	0.0333 (9)	
C33	0.1905 (5)	0.24916 (19)	0.8851 (2)	0.0536 (13)	
H33	0.2598	0.2557	0.9077	0.064*	
C34	0.0911 (6)	0.2796 (2)	0.8809 (3)	0.0660 (17)	
H34	0.0934	0.3066	0.9008	0.079*	
C35	−0.0121 (5)	0.2700 (2)	0.8472 (2)	0.0584 (14)	
H35	−0.0788	0.2907	0.8442	0.070*	
C36	−0.0153 (5)	0.2304 (2)	0.8186 (2)	0.0546 (13)	
H36	−0.0850	0.2238	0.7963	0.066*	
C37	0.0833 (5)	0.19986 (18)	0.82229 (19)	0.0449 (11)	
H37	0.0801	0.1730	0.8021	0.054*	
C38	0.3038 (4)	0.13077 (16)	0.91539 (17)	0.0363 (10)	
H38A	0.3851	0.1182	0.9266	0.044*	
H38B	0.2788	0.1507	0.9426	0.044*	
P4	0.19276 (11)	0.08189 (4)	0.90601 (4)	0.0349 (2)	
C39	0.0385 (4)	0.10805 (16)	0.90295 (18)	0.0381 (10)	
C40	0.0130 (5)	0.14851 (19)	0.9295 (2)	0.0486 (12)	
H40	0.0757	0.1631	0.9504	0.058*	
C41	−0.1067 (6)	0.1673 (2)	0.9248 (2)	0.0588 (15)	
H41	−0.1238	0.1946	0.9423	0.071*	
C42	−0.1978 (6)	0.1457 (2)	0.8945 (3)	0.0642 (16)	
H42	−0.2774	0.1583	0.8913	0.077*	
C43	−0.1744 (5)	0.1052 (2)	0.8683 (3)	0.0646 (16)	
H43	−0.2382	0.0903	0.8483	0.077*	
C44	−0.0551 (5)	0.0869 (2)	0.8721 (2)	0.0521 (13)	
H44	−0.0383	0.0602	0.8536	0.062*	
C45	0.2138 (18)	0.0542 (7)	0.9702 (6)	0.051 (5)	0.443 (16)
C46	0.1644 (17)	0.0725 (6)	1.0117 (5)	0.072 (4)	0.443 (16)
H46	0.1145	0.0990	1.0077	0.086*	0.443 (16)
C47	0.1874 (19)	0.0520 (7)	1.0606 (5)	0.084 (4)	0.443 (16)
H47	0.1524	0.0646	1.0887	0.101*	0.443 (16)
C48	0.260 (2)	0.0147 (8)	1.0659 (7)	0.087 (5)	0.443 (16)
H48	0.2763	0.0016	1.0983	0.104*	0.443 (16)
C49	0.311 (2)	−0.0054 (7)	1.0249 (6)	0.087 (5)	0.443 (16)
H49	0.3585	−0.0325	1.0289	0.105*	0.443 (16)
C50	0.288 (2)	0.0163 (6)	0.9768 (6)	0.074 (4)	0.443 (16)
H50	0.3257	0.0045	0.9489	0.088*	0.443 (16)
C45B	0.2121 (12)	0.0535 (6)	0.9686 (5)	0.051 (4)	0.557 (16)
C46B	0.1177 (13)	0.0481 (5)	0.9986 (5)	0.078 (4)	0.557 (16)
H46B	0.0400	0.0604	0.9879	0.094*	0.557 (16)
C47B	0.1351 (15)	0.0244 (6)	1.0457 (6)	0.093 (4)	0.557 (16)
H47B	0.0696	0.0215	1.0661	0.111*	0.557 (16)
C48B	0.2445 (17)	0.0062 (8)	1.0609 (7)	0.096 (5)	0.557 (16)
H48B	0.2546	−0.0107	1.0914	0.115*	0.557 (16)
C49B	0.3440 (13)	0.0119 (7)	1.0321 (6)	0.092 (4)	0.557 (16)
H49B	0.4226	0.0012	1.0440	0.111*	0.557 (16)
C50B	0.3234 (12)	0.0342 (6)	0.9848 (6)	0.075 (4)	0.557 (16)
H50B	0.3879	0.0359	0.9637	0.091*	0.557 (16)
P5	0.22108 (12)	−0.05512 (4)	0.82795 (5)	0.0405 (3)	
C51	0.2753 (19)	−0.0795 (9)	0.8929 (8)	0.057 (4)	0.443 (16)
C52	0.3948 (19)	−0.0938 (7)	0.9079 (8)	0.062 (4)	0.443 (16)
H52	0.4552	−0.0921	0.8848	0.075*	0.443 (16)
C53	0.4260 (18)	−0.1108 (8)	0.9572 (8)	0.071 (5)	0.443 (16)
H53	0.5068	−0.1205	0.9670	0.085*	0.443 (16)
C54	0.3376 (18)	−0.1132 (8)	0.9911 (7)	0.080 (4)	0.443 (16)
H54	0.3568	−0.1264	1.0233	0.095*	0.443 (16)
C55	0.2229 (16)	−0.0964 (8)	0.9781 (6)	0.081 (4)	0.443 (16)
H55	0.1663	−0.0953	1.0028	0.098*	0.443 (16)
C56	0.1870 (16)	−0.0808 (7)	0.9287 (6)	0.070 (4)	0.443 (16)
H56	0.1057	−0.0714	0.9196	0.085*	0.443 (16)
C51B	0.2798 (15)	−0.0827 (6)	0.8872 (6)	0.052 (3)	0.557 (16)
C52B	0.4062 (15)	−0.0814 (5)	0.9008 (6)	0.057 (3)	0.557 (16)
H52B	0.4589	−0.0671	0.8793	0.068*	0.557 (16)
C53B	0.4541 (16)	−0.1015 (6)	0.9469 (6)	0.073 (4)	0.557 (16)
H53B	0.5390	−0.1009	0.9566	0.087*	0.557 (16)
C54B	0.3749 (18)	−0.1222 (6)	0.9777 (6)	0.083 (4)	0.557 (16)
H54B	0.4074	−0.1358	1.0084	0.099*	0.557 (16)
C55B	0.2512 (16)	−0.1236 (6)	0.9653 (5)	0.090 (4)	0.557 (16)
H55B	0.1997	−0.1372	0.9877	0.108*	0.557 (16)
C56B	0.2001 (14)	−0.1046 (6)	0.9187 (5)	0.075 (3)	0.557 (16)
H56B	0.1153	−0.1065	0.9091	0.089*	0.557 (16)
C57	0.0702 (5)	−0.0826 (2)	0.8134 (2)	0.0506 (12)	
C58	−0.0331 (6)	−0.0548 (3)	0.8076 (3)	0.0708 (17)	
H58	−0.0237	−0.0225	0.8083	0.085*	
C59	−0.1514 (8)	−0.0737 (4)	0.8007 (4)	0.104 (3)	
H59	−0.2208	−0.0544	0.7965	0.124*	
C60	−0.1640 (8)	−0.1210 (4)	0.8003 (4)	0.109 (3)	
H60	−0.2429	−0.1342	0.7981	0.131*	
C61	−0.0614 (9)	−0.1494 (3)	0.8031 (4)	0.113 (3)	
H61	−0.0710	−0.1817	0.8006	0.136*	
C62	0.0550 (8)	−0.1303 (3)	0.8094 (4)	0.093 (3)	
H62	0.1243	−0.1497	0.8110	0.112*	
C63	0.3240 (5)	−0.08141 (16)	0.7838 (2)	0.0453 (11)	
H63A	0.4096	−0.0771	0.7975	0.054*	
H63B	0.3081	−0.1148	0.7814	0.054*	
P6	0.30205 (11)	−0.05569 (4)	0.71930 (5)	0.0373 (2)	
C64	0.1661 (5)	−0.08506 (17)	0.6882 (2)	0.0430 (11)	
C65	0.0617 (5)	−0.05952 (18)	0.6728 (2)	0.0525 (13)	
H65	0.0593	−0.0276	0.6796	0.063*	
C66	−0.0401 (6)	−0.0814 (2)	0.6469 (3)	0.0690 (18)	
H66	−0.1109	−0.0641	0.6366	0.083*	
C67	−0.0373 (6)	−0.1274 (2)	0.6367 (3)	0.074 (2)	
H67	−0.1056	−0.1416	0.6188	0.089*	
C68	0.0650 (6)	−0.1537 (2)	0.6523 (3)	0.0719 (19)	
H68	0.0653	−0.1857	0.6458	0.086*	
C69	0.1676 (5)	−0.13247 (19)	0.6776 (3)	0.0581 (14)	
H69	0.2380	−0.1501	0.6875	0.070*	
C70	0.4268 (5)	−0.07933 (16)	0.6847 (2)	0.0425 (11)	
C71	0.5398 (5)	−0.0934 (2)	0.7075 (3)	0.0614 (15)	
H71	0.5536	−0.0943	0.7431	0.074*	
C72	0.6333 (6)	−0.1064 (3)	0.6777 (3)	0.0739 (19)	
H72	0.7096	−0.1159	0.6936	0.089*	
C73	0.6153 (6)	−0.1055 (2)	0.6256 (3)	0.0665 (17)	
H73	0.6790	−0.1143	0.6060	0.080*	
C74	0.5047 (6)	−0.0918 (2)	0.6024 (2)	0.0643 (16)	
H74	0.4919	−0.0916	0.5667	0.077*	
C75	0.4095 (6)	−0.0780 (2)	0.6315 (2)	0.0561 (14)	
H75	0.3342	−0.0679	0.6152	0.067*	
C76	0.6973 (9)	0.2714 (3)	0.5417 (3)	0.088 (2)	
C77	0.8110 (7)	0.1979 (3)	0.5218 (3)	0.0730 (17)	
C78	0.7947 (8)	0.1618 (3)	0.4878 (3)	0.0845 (19)	
H78	0.7180	0.1578	0.4692	0.101*	
C79	0.8880 (10)	0.1320 (4)	0.4806 (4)	0.102 (2)	
H79	0.8755	0.1075	0.4572	0.122*	
C80	1.0011 (10)	0.1377 (4)	0.5078 (4)	0.105 (2)	
H80	1.0662	0.1174	0.5028	0.126*	
C81	1.0174 (9)	0.1724 (4)	0.5415 (4)	0.101 (2)	
H81	1.0945	0.1760	0.5599	0.121*	
C82	0.9232 (7)	0.2032 (3)	0.5500 (3)	0.0864 (19)	
H82	0.9356	0.2269	0.5743	0.104*	

### Atomic displacement parameters (Å^2^)

**Table:**

	*U*^11^	*U*^22^	*U*^33^	*U*^12^	*U*^13^	*U*^23^
I1	0.03441 (16)	0.04363 (16)	0.04778 (17)	0.00246 (12)	−0.00487 (12)	−0.00638 (12)
I2	0.03447 (16)	0.04649 (17)	0.05071 (18)	−0.00075 (12)	−0.00470 (12)	0.00563 (13)
I3	0.0694 (3)	0.1116 (4)	0.0482 (2)	0.0199 (2)	0.00787 (18)	0.0156 (2)
Ag1	0.0532 (2)	0.02985 (16)	0.04293 (19)	−0.00234 (14)	0.00843 (15)	0.00112 (13)
Ag2	0.0511 (2)	0.03475 (17)	0.03267 (17)	−0.00435 (14)	0.00573 (13)	−0.00388 (12)
Ag3	0.0500 (2)	0.03683 (18)	0.0495 (2)	0.00036 (14)	0.00783 (15)	−0.00519 (14)
S1	0.148 (2)	0.0834 (14)	0.1021 (16)	−0.0216 (14)	0.0263 (15)	0.0202 (12)
N1	0.115 (6)	0.109 (6)	0.097 (5)	0.036 (5)	0.029 (4)	0.034 (4)
N2	0.078 (4)	0.081 (4)	0.075 (4)	0.000 (3)	0.006 (3)	0.010 (3)
P1	0.0388 (6)	0.0306 (5)	0.0325 (5)	−0.0033 (4)	0.0029 (4)	−0.0020 (4)
C1	0.046 (3)	0.036 (2)	0.041 (3)	−0.003 (2)	0.001 (2)	−0.0086 (19)
C2	0.053 (4)	0.099 (5)	0.050 (3)	−0.013 (3)	−0.003 (3)	−0.016 (3)
C3	0.063 (4)	0.125 (7)	0.073 (5)	−0.025 (4)	−0.012 (3)	−0.023 (4)
C4	0.083 (5)	0.107 (6)	0.065 (4)	−0.013 (4)	−0.012 (4)	−0.037 (4)
C5	0.093 (6)	0.140 (8)	0.065 (5)	−0.015 (6)	0.009 (4)	−0.046 (5)
C6	0.071 (4)	0.118 (6)	0.052 (4)	−0.035 (4)	0.015 (3)	−0.036 (4)
C7	0.044 (3)	0.035 (2)	0.033 (2)	−0.0017 (19)	0.0034 (18)	−0.0028 (18)
C8	0.049 (3)	0.048 (3)	0.058 (3)	0.001 (2)	0.010 (2)	0.005 (2)
C9	0.059 (4)	0.071 (4)	0.065 (4)	0.015 (3)	−0.003 (3)	0.002 (3)
C10	0.039 (3)	0.089 (5)	0.064 (4)	0.005 (3)	0.006 (3)	−0.014 (3)
C11	0.047 (3)	0.081 (4)	0.050 (3)	−0.014 (3)	0.010 (2)	0.000 (3)
C12	0.048 (3)	0.055 (3)	0.042 (3)	−0.005 (2)	0.006 (2)	0.003 (2)
C13	0.046 (3)	0.032 (2)	0.031 (2)	−0.0008 (19)	−0.0017 (18)	0.0014 (17)
P2	0.0429 (6)	0.0291 (5)	0.0302 (5)	−0.0023 (4)	0.0024 (4)	−0.0003 (4)
C14	0.050 (3)	0.035 (2)	0.035 (2)	−0.008 (2)	0.001 (2)	−0.0046 (18)
C15	0.049 (3)	0.051 (3)	0.043 (3)	−0.006 (2)	0.005 (2)	0.001 (2)
C16	0.050 (3)	0.079 (4)	0.057 (3)	−0.001 (3)	−0.007 (3)	−0.005 (3)
C17	0.053 (4)	0.105 (5)	0.054 (3)	−0.030 (4)	0.007 (3)	−0.011 (3)
C18	0.074 (4)	0.088 (5)	0.059 (4)	−0.035 (4)	0.011 (3)	0.011 (3)
C19	0.065 (4)	0.057 (3)	0.046 (3)	−0.015 (3)	−0.002 (2)	0.012 (2)
C20	0.055 (3)	0.029 (2)	0.043 (3)	0.001 (2)	0.000 (2)	0.0002 (19)
C21	0.092 (5)	0.047 (3)	0.049 (3)	0.013 (3)	−0.002 (3)	−0.003 (2)
C22	0.111 (6)	0.052 (3)	0.063 (4)	0.017 (4)	−0.019 (4)	0.009 (3)
C23	0.080 (5)	0.038 (3)	0.112 (6)	0.015 (3)	−0.010 (4)	−0.001 (3)
C24	0.089 (5)	0.052 (4)	0.086 (5)	0.020 (3)	0.025 (4)	0.002 (3)
C25	0.083 (4)	0.038 (3)	0.060 (3)	0.010 (3)	0.016 (3)	0.002 (2)
P3	0.0323 (6)	0.0296 (5)	0.0298 (5)	−0.0010 (4)	0.0018 (4)	−0.0039 (4)
C26	0.032 (2)	0.028 (2)	0.040 (2)	0.0036 (17)	−0.0023 (18)	−0.0028 (17)
C27	0.042 (3)	0.039 (2)	0.043 (3)	−0.002 (2)	0.002 (2)	−0.005 (2)
C28	0.041 (3)	0.049 (3)	0.068 (4)	−0.006 (2)	0.011 (2)	0.003 (3)
C29	0.034 (3)	0.042 (3)	0.081 (4)	−0.003 (2)	−0.003 (3)	−0.006 (3)
C30	0.046 (3)	0.051 (3)	0.056 (3)	0.001 (2)	−0.013 (2)	−0.014 (2)
C31	0.046 (3)	0.045 (3)	0.044 (3)	−0.006 (2)	0.001 (2)	−0.009 (2)
C32	0.034 (2)	0.032 (2)	0.033 (2)	0.0006 (17)	0.0051 (17)	−0.0008 (17)
C33	0.050 (3)	0.047 (3)	0.062 (3)	0.005 (2)	−0.003 (2)	−0.018 (2)
C34	0.057 (4)	0.050 (3)	0.090 (5)	0.010 (3)	0.004 (3)	−0.026 (3)
C35	0.048 (3)	0.053 (3)	0.075 (4)	0.017 (3)	0.006 (3)	−0.001 (3)
C36	0.041 (3)	0.065 (3)	0.057 (3)	0.007 (2)	−0.003 (2)	−0.003 (3)
C37	0.049 (3)	0.043 (3)	0.042 (3)	0.002 (2)	0.000 (2)	−0.013 (2)
C38	0.038 (2)	0.037 (2)	0.034 (2)	−0.0023 (19)	0.0022 (18)	−0.0020 (18)
P4	0.0374 (6)	0.0321 (5)	0.0352 (6)	−0.0001 (4)	0.0037 (4)	0.0004 (4)
C39	0.038 (2)	0.038 (2)	0.039 (2)	0.0001 (19)	0.0077 (19)	0.0065 (19)
C40	0.053 (3)	0.047 (3)	0.048 (3)	0.002 (2)	0.014 (2)	0.001 (2)
C41	0.056 (4)	0.053 (3)	0.071 (4)	0.015 (3)	0.023 (3)	0.010 (3)
C42	0.044 (3)	0.069 (4)	0.081 (4)	0.017 (3)	0.012 (3)	0.023 (3)
C43	0.036 (3)	0.073 (4)	0.082 (4)	−0.007 (3)	−0.010 (3)	0.013 (3)
C44	0.047 (3)	0.048 (3)	0.061 (3)	−0.004 (2)	0.002 (2)	0.000 (2)
C45	0.086 (9)	0.040 (7)	0.026 (6)	0.011 (7)	−0.002 (7)	0.001 (6)
C46	0.104 (9)	0.072 (7)	0.040 (6)	0.018 (7)	0.010 (6)	0.012 (5)
C47	0.117 (9)	0.089 (8)	0.045 (6)	0.012 (7)	0.007 (6)	0.019 (6)
C48	0.123 (10)	0.084 (8)	0.048 (7)	0.009 (8)	−0.017 (7)	0.019 (6)
C49	0.122 (10)	0.073 (8)	0.061 (7)	0.020 (8)	−0.022 (7)	0.009 (6)
C50	0.110 (10)	0.062 (7)	0.046 (6)	0.017 (7)	−0.006 (7)	0.000 (6)
C45B	0.037 (5)	0.054 (7)	0.061 (7)	−0.007 (5)	0.004 (5)	0.023 (6)
C46B	0.076 (6)	0.088 (7)	0.073 (7)	0.006 (6)	0.014 (5)	0.035 (6)
C47B	0.100 (7)	0.107 (8)	0.076 (7)	−0.001 (7)	0.027 (6)	0.043 (6)
C48B	0.100 (8)	0.103 (8)	0.084 (8)	−0.009 (7)	0.001 (7)	0.050 (7)
C49B	0.075 (7)	0.100 (8)	0.097 (8)	−0.008 (6)	−0.019 (6)	0.042 (7)
C50B	0.054 (6)	0.083 (7)	0.087 (7)	−0.001 (5)	−0.005 (5)	0.041 (6)
P5	0.0442 (7)	0.0332 (6)	0.0442 (6)	−0.0012 (5)	0.0048 (5)	0.0049 (5)
C51	0.065 (8)	0.056 (8)	0.049 (8)	0.010 (7)	0.007 (7)	0.016 (6)
C52	0.070 (8)	0.067 (9)	0.051 (8)	0.013 (6)	0.012 (7)	0.009 (6)
C53	0.079 (8)	0.082 (9)	0.054 (8)	0.014 (7)	0.013 (7)	0.014 (7)
C54	0.087 (8)	0.095 (9)	0.057 (8)	0.015 (7)	0.012 (7)	0.020 (7)
C55	0.089 (8)	0.094 (9)	0.063 (7)	0.010 (7)	0.018 (6)	0.025 (7)
C56	0.079 (8)	0.075 (8)	0.059 (7)	0.005 (6)	0.015 (6)	0.029 (6)
C51B	0.068 (7)	0.045 (6)	0.042 (6)	−0.006 (5)	−0.002 (5)	0.005 (5)
C52B	0.074 (7)	0.045 (6)	0.049 (6)	0.007 (5)	−0.009 (5)	0.009 (5)
C53B	0.094 (8)	0.068 (6)	0.053 (7)	0.013 (6)	−0.011 (6)	0.014 (5)
C54B	0.109 (8)	0.083 (7)	0.054 (7)	0.012 (6)	−0.009 (7)	0.022 (6)
C55B	0.117 (8)	0.089 (7)	0.062 (6)	−0.006 (7)	0.005 (6)	0.027 (6)
C56B	0.091 (7)	0.075 (7)	0.058 (6)	−0.009 (6)	0.007 (5)	0.019 (5)
C57	0.051 (3)	0.055 (3)	0.047 (3)	−0.009 (2)	0.005 (2)	0.007 (2)
C58	0.056 (4)	0.076 (4)	0.080 (4)	−0.005 (3)	0.003 (3)	−0.010 (3)
C59	0.057 (5)	0.125 (8)	0.127 (8)	−0.004 (5)	−0.003 (4)	−0.010 (6)
C60	0.064 (5)	0.144 (9)	0.116 (7)	−0.049 (6)	−0.003 (5)	0.014 (6)
C61	0.099 (7)	0.082 (6)	0.154 (9)	−0.047 (5)	−0.021 (6)	0.023 (6)
C62	0.079 (5)	0.053 (4)	0.143 (8)	−0.022 (4)	−0.013 (5)	0.029 (4)
C63	0.053 (3)	0.031 (2)	0.053 (3)	0.000 (2)	0.005 (2)	0.002 (2)
P6	0.0394 (6)	0.0265 (5)	0.0462 (6)	−0.0036 (4)	0.0056 (5)	−0.0022 (4)
C64	0.043 (3)	0.036 (2)	0.051 (3)	−0.006 (2)	0.007 (2)	−0.004 (2)
C65	0.046 (3)	0.037 (3)	0.075 (4)	−0.004 (2)	0.009 (3)	−0.014 (2)
C66	0.044 (3)	0.059 (4)	0.103 (5)	0.000 (3)	−0.003 (3)	−0.014 (3)
C67	0.046 (3)	0.062 (4)	0.115 (6)	−0.012 (3)	0.004 (3)	−0.030 (4)
C68	0.056 (4)	0.047 (3)	0.114 (6)	−0.017 (3)	0.013 (3)	−0.030 (3)
C69	0.049 (3)	0.036 (3)	0.089 (4)	−0.005 (2)	0.005 (3)	−0.005 (3)
C70	0.045 (3)	0.030 (2)	0.053 (3)	−0.0053 (19)	0.009 (2)	−0.006 (2)
C71	0.042 (3)	0.076 (4)	0.066 (4)	0.004 (3)	0.006 (3)	−0.007 (3)
C72	0.046 (4)	0.090 (5)	0.087 (5)	0.006 (3)	0.009 (3)	−0.007 (4)
C73	0.054 (4)	0.074 (4)	0.075 (4)	−0.005 (3)	0.025 (3)	−0.021 (3)
C74	0.067 (4)	0.071 (4)	0.057 (3)	−0.011 (3)	0.018 (3)	−0.014 (3)
C75	0.051 (3)	0.058 (3)	0.061 (3)	0.001 (3)	0.008 (3)	−0.004 (3)
C76	0.109 (6)	0.096 (6)	0.061 (4)	0.014 (5)	0.019 (4)	0.030 (4)
C77	0.073 (4)	0.081 (4)	0.066 (4)	0.001 (3)	0.011 (3)	0.025 (3)
C78	0.094 (5)	0.092 (5)	0.067 (4)	0.012 (4)	0.002 (3)	0.013 (3)
C79	0.113 (5)	0.102 (5)	0.092 (5)	0.009 (5)	0.014 (4)	0.011 (4)
C80	0.098 (5)	0.108 (5)	0.112 (6)	0.012 (5)	0.029 (4)	0.020 (5)
C81	0.077 (4)	0.113 (5)	0.113 (5)	−0.002 (4)	0.007 (4)	0.016 (5)
C82	0.073 (4)	0.095 (5)	0.090 (4)	−0.005 (4)	0.004 (4)	0.013 (4)

### Geometric parameters (Å, º)

**Table:**

I1—Ag1	2.8975 (5)	C40—C41	1.397 (8)
I1—Ag2	2.9687 (5)	C40—H40	0.9300
I1—Ag3	2.9916 (5)	C41—C42	1.356 (9)
I2—Ag3	2.9686 (5)	C41—H41	0.9300
I2—Ag1	2.9711 (5)	C42—C43	1.378 (10)
I2—Ag2	3.0455 (5)	C42—H42	0.9300
Ag1—P6	2.4614 (11)	C43—C44	1.388 (8)
Ag1—P1	2.4621 (12)	C43—H43	0.9300
Ag1—Ag2	3.0823 (5)	C44—H44	0.9300
Ag1—Ag3	3.2026 (5)	C45—C50	1.350 (15)
Ag2—P3	2.4690 (11)	C45—C46	1.357 (15)
Ag2—P2	2.4836 (11)	C46—C47	1.408 (15)
Ag2—Ag3	3.2999 (5)	C46—H46	0.9300
Ag3—P4	2.4679 (12)	C47—C48	1.321 (18)
Ag3—P5	2.4753 (12)	C47—H47	0.9300
S1—C76	1.651 (10)	C48—C49	1.378 (19)
N1—C76	1.355 (11)	C48—H48	0.9300
N1—H1A	0.8600	C49—C50	1.404 (15)
N1—H1B	0.8600	C49—H49	0.9300
N2—C76	1.343 (11)	C50—H50	0.9300
N2—C77	1.432 (10)	C45B—C46B	1.354 (14)
N2—H2A	0.8600	C45B—C50B	1.356 (13)
P1—C7	1.814 (5)	C46B—C47B	1.403 (14)
P1—C1	1.836 (5)	C46B—H46B	0.9300
P1—C13	1.838 (4)	C47B—C48B	1.320 (17)
C1—C6	1.359 (8)	C47B—H47B	0.9300
C1—C2	1.373 (8)	C48B—C49B	1.379 (18)
C2—C3	1.380 (9)	C48B—H48B	0.9300
C2—H2	0.9300	C49B—C50B	1.393 (14)
C3—C4	1.368 (11)	C49B—H49B	0.9300
C3—H3	0.9300	C50B—H50B	0.9300
C4—C5	1.324 (11)	P5—C51B	1.803 (15)
C4—H4	0.9300	P5—C57	1.819 (6)
C5—C6	1.388 (9)	P5—C63	1.838 (5)
C5—H5	0.9300	P5—C51	1.879 (19)
C6—H6	0.9300	C51—C52	1.378 (15)
C7—C8	1.386 (7)	C51—C56	1.400 (16)
C7—C12	1.395 (7)	C52—C53	1.390 (16)
C8—C9	1.385 (9)	C52—H52	0.9300
C8—H8	0.9300	C53—C54	1.365 (19)
C9—C10	1.363 (10)	C53—H53	0.9300
C9—H9	0.9300	C54—C55	1.343 (19)
C10—C11	1.366 (9)	C54—H54	0.9300
C10—H10	0.9300	C55—C56	1.389 (15)
C11—C12	1.382 (8)	C55—H55	0.9300
C11—H11	0.9300	C56—H56	0.9300
C12—H12	0.9300	C51B—C52B	1.381 (13)
C13—P2	1.838 (4)	C51B—C56B	1.394 (14)
C13—H13A	0.9700	C52B—C53B	1.393 (13)
C13—H13B	0.9700	C52B—H52B	0.9300
P2—C14	1.824 (5)	C53B—C54B	1.363 (18)
P2—C20	1.832 (5)	C53B—H53B	0.9300
C14—C19	1.387 (7)	C54B—C55B	1.349 (19)
C14—C15	1.393 (7)	C54B—H54B	0.9300
C15—C16	1.380 (8)	C55B—C56B	1.400 (14)
C15—H15	0.9300	C55B—H55B	0.9300
C16—C17	1.371 (10)	C56B—H56B	0.9300
C16—H16	0.9300	C57—C58	1.366 (9)
C17—C18	1.373 (10)	C57—C62	1.376 (9)
C17—H17	0.9300	C58—C59	1.385 (11)
C18—C19	1.373 (9)	C58—H58	0.9300
C18—H18	0.9300	C59—C60	1.358 (14)
C19—H19	0.9300	C59—H59	0.9300
C20—C21	1.380 (8)	C60—C61	1.371 (14)
C20—C25	1.383 (8)	C60—H60	0.9300
C21—C22	1.378 (9)	C61—C62	1.368 (11)
C21—H21	0.9300	C61—H61	0.9300
C22—C23	1.388 (11)	C62—H62	0.9300
C22—H22	0.9300	C63—P6	1.835 (5)
C23—C24	1.350 (10)	C63—H63A	0.9700
C23—H23	0.9300	C63—H63B	0.9700
C24—C25	1.403 (9)	P6—C64	1.818 (5)
C24—H24	0.9300	P6—C70	1.824 (5)
C25—H25	0.9300	C64—C65	1.373 (8)
P3—C26	1.822 (4)	C64—C69	1.383 (7)
P3—C32	1.837 (4)	C65—C66	1.388 (8)
P3—C38	1.845 (5)	C65—H65	0.9300
C26—C27	1.390 (7)	C66—C67	1.342 (9)
C26—C31	1.400 (6)	C66—H66	0.9300
C27—C28	1.385 (7)	C67—C68	1.366 (10)
C27—H27	0.9300	C67—H67	0.9300
C28—C29	1.363 (8)	C68—C69	1.378 (8)
C28—H28	0.9300	C68—H68	0.9300
C29—C30	1.377 (9)	C69—H69	0.9300
C29—H29	0.9300	C70—C71	1.370 (8)
C30—C31	1.386 (7)	C70—C75	1.388 (8)
C30—H30	0.9300	C71—C72	1.382 (9)
C31—H31	0.9300	C71—H71	0.9300
C32—C37	1.377 (6)	C72—C73	1.360 (10)
C32—C33	1.390 (7)	C72—H72	0.9300
C33—C34	1.379 (8)	C73—C74	1.348 (10)
C33—H33	0.9300	C73—H73	0.9300
C34—C35	1.384 (9)	C74—C75	1.392 (9)
C34—H34	0.9300	C74—H74	0.9300
C35—C36	1.356 (8)	C75—H75	0.9300
C35—H35	0.9300	C77—C78	1.362 (11)
C36—C37	1.374 (8)	C77—C82	1.369 (10)
C36—H36	0.9300	C78—C79	1.348 (12)
C37—H37	0.9300	C78—H78	0.9300
C38—P4	1.843 (5)	C79—C80	1.368 (14)
C38—H38A	0.9700	C79—H79	0.9300
C38—H38B	0.9700	C80—C81	1.327 (14)
P4—C45B	1.821 (13)	C80—H80	0.9300
P4—C39	1.823 (5)	C81—C82	1.380 (13)
P4—C45	1.852 (15)	C81—H81	0.9300
C39—C44	1.375 (7)	C82—H82	0.9300
C39—C40	1.389 (7)		
			
Ag1—I1—Ag2	63.383 (11)	C45B—P4—C39	104.5 (4)
Ag1—I1—Ag3	65.867 (12)	C45B—P4—C38	101.4 (6)
Ag2—I1—Ag3	67.235 (11)	C39—P4—C38	106.1 (2)
Ag3—I2—Ag1	65.257 (12)	C39—P4—C45	104.5 (5)
Ag3—I2—Ag2	66.541 (12)	C38—P4—C45	100.3 (7)
Ag1—I2—Ag2	61.620 (10)	C45B—P4—Ag3	115.3 (5)
P6—Ag1—P1	117.77 (4)	C39—P4—Ag3	114.90 (16)
P6—Ag1—I1	112.01 (3)	C38—P4—Ag3	113.25 (15)
P1—Ag1—I1	107.39 (3)	C45—P4—Ag3	116.2 (5)
P6—Ag1—I2	100.87 (3)	C44—C39—C40	119.3 (5)
P1—Ag1—I2	112.89 (3)	C44—C39—P4	117.8 (4)
I1—Ag1—I2	105.217 (14)	C40—C39—P4	122.9 (4)
P6—Ag1—Ag2	151.56 (3)	C39—C40—C41	120.2 (5)
P1—Ag1—Ag2	90.20 (3)	C39—C40—H40	119.9
I1—Ag1—Ag2	59.434 (11)	C41—C40—H40	119.9
I2—Ag1—Ag2	60.377 (11)	C42—C41—C40	119.5 (6)
P6—Ag1—Ag3	88.81 (3)	C42—C41—H41	120.2
P1—Ag1—Ag3	153.41 (3)	C40—C41—H41	120.2
I1—Ag1—Ag3	58.479 (11)	C41—C42—C43	121.1 (5)
I2—Ag1—Ag3	57.334 (12)	C41—C42—H42	119.5
Ag2—Ag1—Ag3	63.309 (11)	C43—C42—H42	119.5
P3—Ag2—P2	123.75 (4)	C42—C43—C44	119.5 (6)
P3—Ag2—I1	111.95 (3)	C42—C43—H43	120.2
P2—Ag2—I1	102.45 (3)	C44—C43—H43	120.2
P3—Ag2—I2	99.29 (3)	C39—C44—C43	120.4 (6)
P2—Ag2—I2	116.00 (3)	C39—C44—H44	119.8
I1—Ag2—I2	101.661 (13)	C43—C44—H44	119.8
P3—Ag2—Ag1	146.55 (3)	C50—C45—C46	118.8 (13)
P2—Ag2—Ag1	89.53 (3)	C50—C45—P4	118.7 (11)
I1—Ag2—Ag1	57.183 (11)	C46—C45—P4	122.4 (11)
I2—Ag2—Ag1	58.003 (11)	C45—C46—C47	121.2 (13)
P3—Ag2—Ag3	87.10 (3)	C45—C46—H46	119.4
P2—Ag2—Ag3	148.86 (3)	C47—C46—H46	119.4
I1—Ag2—Ag3	56.713 (11)	C48—C47—C46	118.8 (14)
I2—Ag2—Ag3	55.614 (11)	C48—C47—H47	120.6
Ag1—Ag2—Ag3	60.124 (11)	C46—C47—H47	120.6
P4—Ag3—P5	127.78 (4)	C47—C48—C49	122.2 (14)
P4—Ag3—I2	106.61 (3)	C47—C48—H48	118.9
P5—Ag3—I2	98.37 (3)	C49—C48—H48	118.9
P4—Ag3—I1	105.83 (3)	C48—C49—C50	117.6 (14)
P5—Ag3—I1	112.23 (3)	C48—C49—H49	121.2
I2—Ag3—I1	102.950 (14)	C50—C49—H49	121.2
P4—Ag3—Ag1	144.31 (3)	C45—C50—C49	121.3 (14)
P5—Ag3—Ag1	87.64 (3)	C45—C50—H50	119.3
I2—Ag3—Ag1	57.409 (11)	C49—C50—H50	119.3
I1—Ag3—Ag1	55.654 (11)	C46B—C45B—C50B	117.9 (12)
P4—Ag3—Ag2	87.76 (3)	C46B—C45B—P4	122.8 (10)
P5—Ag3—Ag2	143.41 (3)	C50B—C45B—P4	119.1 (9)
I2—Ag3—Ag2	57.845 (11)	C45B—C46B—C47B	121.1 (12)
I1—Ag3—Ag2	56.051 (11)	C45B—C46B—H46B	119.4
Ag1—Ag3—Ag2	56.567 (10)	C47B—C46B—H46B	119.4
C76—N1—H1A	120.0	C48B—C47B—C46B	120.0 (13)
C76—N1—H1B	120.0	C48B—C47B—H47B	120.0
H1A—N1—H1B	120.0	C46B—C47B—H47B	120.0
C76—N2—C77	129.6 (7)	C47B—C48B—C49B	120.8 (13)
C76—N2—H2A	115.2	C47B—C48B—H48B	119.6
C77—N2—H2A	115.2	C49B—C48B—H48B	119.6
C7—P1—C1	106.0 (2)	C48B—C49B—C50B	118.1 (12)
C7—P1—C13	106.9 (2)	C48B—C49B—H49B	120.9
C1—P1—C13	101.2 (2)	C50B—C49B—H49B	120.9
C7—P1—Ag1	117.36 (15)	C45B—C50B—C49B	121.9 (12)
C1—P1—Ag1	111.52 (17)	C45B—C50B—H50B	119.1
C13—P1—Ag1	112.43 (16)	C49B—C50B—H50B	119.1
C6—C1—C2	118.3 (5)	C51B—P5—C57	103.3 (5)
C6—C1—P1	124.9 (4)	C51B—P5—C63	100.2 (6)
C2—C1—P1	116.8 (4)	C57—P5—C63	106.0 (3)
C1—C2—C3	120.6 (6)	C57—P5—C51	103.2 (7)
C1—C2—H2	119.7	C63—P5—C51	105.1 (7)
C3—C2—H2	119.7	C51B—P5—Ag3	112.7 (6)
C4—C3—C2	119.8 (7)	C57—P5—Ag3	118.11 (19)
C4—C3—H3	120.1	C63—P5—Ag3	114.48 (16)
C2—C3—H3	120.1	C51—P5—Ag3	108.6 (8)
C5—C4—C3	119.8 (6)	C52—C51—C56	118.8 (14)
C5—C4—H4	120.1	C52—C51—P5	125.0 (12)
C3—C4—H4	120.1	C56—C51—P5	116.1 (13)
C4—C5—C6	121.1 (7)	C51—C52—C53	120.6 (14)
C4—C5—H5	119.4	C51—C52—H52	119.7
C6—C5—H5	119.4	C53—C52—H52	119.7
C1—C6—C5	120.3 (7)	C54—C53—C52	119.7 (15)
C1—C6—H6	119.8	C54—C53—H53	120.2
C5—C6—H6	119.8	C52—C53—H53	120.2
C8—C7—C12	118.2 (5)	C55—C54—C53	120.4 (15)
C8—C7—P1	117.5 (4)	C55—C54—H54	119.8
C12—C7—P1	124.3 (4)	C53—C54—H54	119.8
C9—C8—C7	121.2 (5)	C54—C55—C56	121.4 (15)
C9—C8—H8	119.4	C54—C55—H55	119.3
C7—C8—H8	119.4	C56—C55—H55	119.3
C10—C9—C8	119.1 (6)	C55—C56—C51	118.9 (14)
C10—C9—H9	120.5	C55—C56—H56	120.6
C8—C9—H9	120.5	C51—C56—H56	120.6
C9—C10—C11	121.4 (6)	C52B—C51B—C56B	120.9 (12)
C9—C10—H10	119.3	C52B—C51B—P5	118.0 (10)
C11—C10—H10	119.3	C56B—C51B—P5	121.1 (11)
C10—C11—C12	119.8 (6)	C51B—C52B—C53B	119.4 (13)
C10—C11—H11	120.1	C51B—C52B—H52B	120.3
C12—C11—H11	120.1	C53B—C52B—H52B	120.3
C11—C12—C7	120.3 (5)	C54B—C53B—C52B	119.2 (13)
C11—C12—H12	119.8	C54B—C53B—H53B	120.4
C7—C12—H12	119.8	C52B—C53B—H53B	120.4
P1—C13—P2	113.7 (2)	C55B—C54B—C53B	122.3 (12)
P1—C13—H13A	108.8	C55B—C54B—H54B	118.9
P2—C13—H13A	108.8	C53B—C54B—H54B	118.9
P1—C13—H13B	108.8	C54B—C55B—C56B	120.1 (13)
P2—C13—H13B	108.8	C54B—C55B—H55B	120.0
H13A—C13—H13B	107.7	C56B—C55B—H55B	120.0
C14—P2—C20	107.4 (2)	C51B—C56B—C55B	118.2 (13)
C14—P2—C13	104.7 (2)	C51B—C56B—H56B	120.9
C20—P2—C13	101.0 (2)	C55B—C56B—H56B	120.9
C14—P2—Ag2	115.15 (15)	C58—C57—C62	118.4 (6)
C20—P2—Ag2	112.91 (17)	C58—C57—P5	118.8 (5)
C13—P2—Ag2	114.36 (15)	C62—C57—P5	122.7 (5)
C19—C14—C15	118.5 (5)	C57—C58—C59	121.7 (8)
C19—C14—P2	124.2 (4)	C57—C58—H58	119.2
C15—C14—P2	117.3 (4)	C59—C58—H58	119.2
C16—C15—C14	120.1 (5)	C60—C59—C58	118.6 (9)
C16—C15—H15	120.0	C60—C59—H59	120.7
C14—C15—H15	120.0	C58—C59—H59	120.7
C17—C16—C15	120.8 (6)	C59—C60—C61	120.5 (8)
C17—C16—H16	119.6	C59—C60—H60	119.8
C15—C16—H16	119.6	C61—C60—H60	119.8
C16—C17—C18	119.4 (6)	C62—C61—C60	120.2 (9)
C16—C17—H17	120.3	C62—C61—H61	119.9
C18—C17—H17	120.3	C60—C61—H61	119.9
C17—C18—C19	120.7 (6)	C61—C62—C57	120.3 (8)
C17—C18—H18	119.7	C61—C62—H62	119.8
C19—C18—H18	119.7	C57—C62—H62	119.8
C18—C19—C14	120.6 (6)	P6—C63—P5	112.3 (3)
C18—C19—H19	119.7	P6—C63—H63A	109.1
C14—C19—H19	119.7	P5—C63—H63A	109.1
C21—C20—C25	118.3 (5)	P6—C63—H63B	109.1
C21—C20—P2	123.4 (4)	P5—C63—H63B	109.1
C25—C20—P2	118.1 (4)	H63A—C63—H63B	107.9
C22—C21—C20	121.2 (6)	C64—P6—C70	102.2 (2)
C22—C21—H21	119.4	C64—P6—C63	104.9 (2)
C20—C21—H21	119.4	C70—P6—C63	105.7 (2)
C21—C22—C23	119.9 (6)	C64—P6—Ag1	122.41 (17)
C21—C22—H22	120.1	C70—P6—Ag1	107.26 (15)
C23—C22—H22	120.1	C63—P6—Ag1	112.84 (16)
C24—C23—C22	119.8 (6)	C65—C64—C69	119.0 (5)
C24—C23—H23	120.1	C65—C64—P6	119.7 (4)
C22—C23—H23	120.1	C69—C64—P6	121.2 (4)
C23—C24—C25	120.5 (6)	C64—C65—C66	119.8 (5)
C23—C24—H24	119.7	C64—C65—H65	120.1
C25—C24—H24	119.7	C66—C65—H65	120.1
C20—C25—C24	120.2 (6)	C67—C66—C65	120.5 (6)
C20—C25—H25	119.9	C67—C66—H66	119.8
C24—C25—H25	119.9	C65—C66—H66	119.8
C26—P3—C32	104.3 (2)	C66—C67—C68	120.7 (6)
C26—P3—C38	103.0 (2)	C66—C67—H67	119.7
C32—P3—C38	105.8 (2)	C68—C67—H67	119.7
C26—P3—Ag2	111.72 (15)	C67—C68—C69	119.7 (6)
C32—P3—Ag2	113.43 (14)	C67—C68—H68	120.1
C38—P3—Ag2	117.28 (15)	C69—C68—H68	120.1
C27—C26—C31	118.3 (4)	C68—C69—C64	120.2 (6)
C27—C26—P3	117.9 (3)	C68—C69—H69	119.9
C31—C26—P3	123.8 (4)	C64—C69—H69	119.9
C28—C27—C26	120.7 (5)	C71—C70—C75	118.5 (5)
C28—C27—H27	119.6	C71—C70—P6	124.3 (4)
C26—C27—H27	119.6	C75—C70—P6	116.8 (4)
C29—C28—C27	120.7 (5)	C70—C71—C72	120.2 (6)
C29—C28—H28	119.7	C70—C71—H71	119.9
C27—C28—H28	119.7	C72—C71—H71	119.9
C28—C29—C30	119.6 (5)	C73—C72—C71	121.0 (6)
C28—C29—H29	120.2	C73—C72—H72	119.5
C30—C29—H29	120.2	C71—C72—H72	119.5
C29—C30—C31	120.9 (5)	C74—C73—C72	119.7 (6)
C29—C30—H30	119.6	C74—C73—H73	120.2
C31—C30—H30	119.6	C72—C73—H73	120.2
C30—C31—C26	119.9 (5)	C73—C74—C75	120.4 (6)
C30—C31—H31	120.1	C73—C74—H74	119.8
C26—C31—H31	120.1	C75—C74—H74	119.8
C37—C32—C33	118.8 (4)	C70—C75—C74	120.2 (6)
C37—C32—P3	118.2 (3)	C70—C75—H75	119.9
C33—C32—P3	122.9 (4)	C74—C75—H75	119.9
C34—C33—C32	119.9 (5)	N2—C76—N1	111.2 (8)
C34—C33—H33	120.0	N2—C76—S1	127.4 (7)
C32—C33—H33	120.0	N1—C76—S1	121.4 (8)
C33—C34—C35	120.2 (5)	C78—C77—C82	119.3 (8)
C33—C34—H34	119.9	C78—C77—N2	116.5 (7)
C35—C34—H34	119.9	C82—C77—N2	124.1 (8)
C36—C35—C34	119.6 (5)	C79—C78—C77	121.2 (9)
C36—C35—H35	120.2	C79—C78—H78	119.4
C34—C35—H35	120.2	C77—C78—H78	119.4
C35—C36—C37	120.8 (5)	C78—C79—C80	119.8 (10)
C35—C36—H36	119.6	C78—C79—H79	120.1
C37—C36—H36	119.6	C80—C79—H79	120.1
C36—C37—C32	120.6 (5)	C81—C80—C79	119.5 (10)
C36—C37—H37	119.7	C81—C80—H80	120.3
C32—C37—H37	119.7	C79—C80—H80	120.3
P4—C38—P3	114.2 (2)	C80—C81—C82	121.9 (10)
P4—C38—H38A	108.7	C80—C81—H81	119.0
P3—C38—H38A	108.7	C82—C81—H81	119.0
P4—C38—H38B	108.7	C77—C82—C81	118.3 (9)
P3—C38—H38B	108.7	C77—C82—H82	120.8
H38A—C38—H38B	107.6	C81—C82—H82	120.8
			
C7—P1—C1—C6	−19.0 (6)	C39—P4—C45—C46	−33.3 (17)
C13—P1—C1—C6	92.4 (6)	C38—P4—C45—C46	76.5 (16)
Ag1—P1—C1—C6	−147.9 (6)	Ag3—P4—C45—C46	−161.1 (13)
C7—P1—C1—C2	159.3 (5)	C50—C45—C46—C47	−1.5 (19)
C13—P1—C1—C2	−89.3 (5)	P4—C45—C46—C47	−176.6 (16)
Ag1—P1—C1—C2	30.4 (5)	C45—C46—C47—C48	1 (2)
C6—C1—C2—C3	1.6 (11)	C46—C47—C48—C49	−1 (3)
P1—C1—C2—C3	−176.8 (6)	C47—C48—C49—C50	3 (4)
C1—C2—C3—C4	−1.2 (13)	C46—C45—C50—C49	3 (3)
C2—C3—C4—C5	1.3 (15)	P4—C45—C50—C49	178.2 (16)
C3—C4—C5—C6	−2.0 (16)	C48—C49—C50—C45	−4 (3)
C2—C1—C6—C5	−2.3 (12)	C39—P4—C45B—C46B	10.3 (15)
P1—C1—C6—C5	176.0 (7)	C38—P4—C45B—C46B	120.5 (13)
C4—C5—C6—C1	2.5 (16)	Ag3—P4—C45B—C46B	−116.7 (12)
C1—P1—C7—C8	−107.6 (4)	C39—P4—C45B—C50B	−173.9 (14)
C13—P1—C7—C8	145.1 (4)	C38—P4—C45B—C50B	−63.7 (15)
Ag1—P1—C7—C8	17.8 (4)	Ag3—P4—C45B—C50B	59.0 (16)
C1—P1—C7—C12	73.3 (5)	C50B—C45B—C46B—C47B	1.5 (17)
C13—P1—C7—C12	−34.1 (5)	P4—C45B—C46B—C47B	177.4 (13)
Ag1—P1—C7—C12	−161.3 (4)	C45B—C46B—C47B—C48B	−1.1 (19)
C12—C7—C8—C9	1.0 (8)	C46B—C47B—C48B—C49B	3 (3)
P1—C7—C8—C9	−178.2 (5)	C47B—C48B—C49B—C50B	−5 (3)
C7—C8—C9—C10	−0.7 (9)	C46B—C45B—C50B—C49B	−4 (2)
C8—C9—C10—C11	−0.1 (10)	P4—C45B—C50B—C49B	−179.8 (14)
C9—C10—C11—C12	0.7 (10)	C48B—C49B—C50B—C45B	5 (3)
C10—C11—C12—C7	−0.4 (9)	C57—P5—C51—C52	138.9 (17)
C8—C7—C12—C11	−0.4 (8)	C63—P5—C51—C52	28 (2)
P1—C7—C12—C11	178.7 (4)	Ag3—P5—C51—C52	−94.9 (18)
C7—P1—C13—P2	−79.3 (3)	C57—P5—C51—C56	−44 (2)
C1—P1—C13—P2	170.0 (3)	C63—P5—C51—C56	−155.2 (17)
Ag1—P1—C13—P2	50.9 (3)	Ag3—P5—C51—C56	81.9 (19)
P1—C13—P2—C14	81.1 (3)	C56—C51—C52—C53	2.3 (19)
P1—C13—P2—C20	−167.4 (3)	P5—C51—C52—C53	179 (2)
P1—C13—P2—Ag2	−45.8 (3)	C51—C52—C53—C54	−0.3 (18)
C20—P2—C14—C19	−27.0 (5)	C52—C53—C54—C55	−4 (3)
C13—P2—C14—C19	79.9 (5)	C53—C54—C55—C56	7 (3)
Ag2—P2—C14—C19	−153.7 (4)	C54—C55—C56—C51	−5 (3)
C20—P2—C14—C15	154.7 (4)	C52—C51—C56—C55	0 (3)
C13—P2—C14—C15	−98.5 (4)	P5—C51—C56—C55	−177.0 (16)
Ag2—P2—C14—C15	28.0 (4)	C57—P5—C51B—C52B	159.8 (11)
C19—C14—C15—C16	−2.6 (8)	C63—P5—C51B—C52B	50.5 (12)
P2—C14—C15—C16	175.8 (4)	Ag3—P5—C51B—C52B	−71.7 (12)
C14—C15—C16—C17	2.2 (9)	C57—P5—C51B—C56B	−20.7 (16)
C15—C16—C17—C18	−0.4 (10)	C63—P5—C51B—C56B	−130.0 (14)
C16—C17—C18—C19	−1.0 (11)	Ag3—P5—C51B—C56B	107.9 (14)
C17—C18—C19—C14	0.5 (10)	C56B—C51B—C52B—C53B	−1.0 (16)
C15—C14—C19—C18	1.3 (8)	P5—C51B—C52B—C53B	178.6 (13)
P2—C14—C19—C18	−177.0 (5)	C51B—C52B—C53B—C54B	0.0 (15)
C14—P2—C20—C21	77.9 (5)	C52B—C53B—C54B—C55B	0 (3)
C13—P2—C20—C21	−31.4 (6)	C53B—C54B—C55B—C56B	2 (3)
Ag2—P2—C20—C21	−154.0 (5)	C52B—C51B—C56B—C55B	2 (2)
C14—P2—C20—C25	−107.5 (5)	P5—C51B—C56B—C55B	−177.3 (13)
C13—P2—C20—C25	143.2 (5)	C54B—C55B—C56B—C51B	−3 (3)
Ag2—P2—C20—C25	20.6 (5)	C51B—P5—C57—C58	122.4 (8)
C25—C20—C21—C22	−1.3 (10)	C63—P5—C57—C58	−132.7 (5)
P2—C20—C21—C22	173.2 (5)	C51—P5—C57—C58	117.1 (9)
C20—C21—C22—C23	−0.9 (11)	Ag3—P5—C57—C58	−2.7 (6)
C21—C22—C23—C24	1.5 (12)	C51B—P5—C57—C62	−55.6 (9)
C22—C23—C24—C25	0.2 (12)	C63—P5—C57—C62	49.3 (7)
C21—C20—C25—C24	3.0 (9)	C51—P5—C57—C62	−60.9 (10)
P2—C20—C25—C24	−171.8 (5)	Ag3—P5—C57—C62	179.2 (6)
C23—C24—C25—C20	−2.5 (11)	C62—C57—C58—C59	3.6 (11)
C32—P3—C26—C27	−99.9 (4)	P5—C57—C58—C59	−174.6 (7)
C38—P3—C26—C27	149.8 (4)	C57—C58—C59—C60	0.7 (14)
Ag2—P3—C26—C27	23.0 (4)	C58—C59—C60—C61	−4.4 (16)
C32—P3—C26—C31	79.3 (4)	C59—C60—C61—C62	3.9 (17)
C38—P3—C26—C31	−31.1 (4)	C60—C61—C62—C57	0.5 (16)
Ag2—P3—C26—C31	−157.8 (4)	C58—C57—C62—C61	−4.1 (13)
C31—C26—C27—C28	−1.0 (7)	P5—C57—C62—C61	173.9 (7)
P3—C26—C27—C28	178.2 (4)	C51B—P5—C63—P6	−173.4 (6)
C26—C27—C28—C29	1.3 (8)	C57—P5—C63—P6	79.5 (3)
C27—C28—C29—C30	−1.0 (8)	C51—P5—C63—P6	−171.6 (8)
C28—C29—C30—C31	0.4 (8)	Ag3—P5—C63—P6	−52.6 (3)
C29—C30—C31—C26	−0.2 (8)	P5—C63—P6—C64	−81.0 (3)
C27—C26—C31—C30	0.5 (7)	P5—C63—P6—C70	171.4 (3)
P3—C26—C31—C30	−178.7 (4)	P5—C63—P6—Ag1	54.5 (3)
C26—P3—C32—C37	150.1 (4)	C70—P6—C64—C65	−132.3 (5)
C38—P3—C32—C37	−101.6 (4)	C63—P6—C64—C65	117.6 (5)
Ag2—P3—C32—C37	28.3 (4)	Ag1—P6—C64—C65	−12.5 (5)
C26—P3—C32—C33	−28.1 (5)	C70—P6—C64—C69	44.6 (5)
C38—P3—C32—C33	80.2 (5)	C63—P6—C64—C69	−65.5 (5)
Ag2—P3—C32—C33	−149.9 (4)	Ag1—P6—C64—C69	164.4 (4)
C37—C32—C33—C34	0.3 (9)	C69—C64—C65—C66	0.0 (9)
P3—C32—C33—C34	178.5 (5)	P6—C64—C65—C66	177.0 (5)
C32—C33—C34—C35	−0.3 (10)	C64—C65—C66—C67	−0.3 (11)
C33—C34—C35—C36	0.5 (11)	C65—C66—C67—C68	1.1 (13)
C34—C35—C36—C37	−0.8 (10)	C66—C67—C68—C69	−1.7 (12)
C35—C36—C37—C32	0.8 (9)	C67—C68—C69—C64	1.5 (11)
C33—C32—C37—C36	−0.6 (8)	C65—C64—C69—C68	−0.6 (10)
P3—C32—C37—C36	−178.9 (4)	P6—C64—C69—C68	−177.6 (5)
C26—P3—C38—P4	−167.9 (2)	C64—P6—C70—C71	−137.1 (5)
C32—P3—C38—P4	82.9 (3)	C63—P6—C70—C71	−27.6 (5)
Ag2—P3—C38—P4	−44.8 (3)	Ag1—P6—C70—C71	93.0 (5)
P3—C38—P4—C45B	177.1 (5)	C64—P6—C70—C75	50.0 (4)
P3—C38—P4—C39	−73.9 (3)	C63—P6—C70—C75	159.5 (4)
P3—C38—P4—C45	177.6 (5)	Ag1—P6—C70—C75	−79.9 (4)
P3—C38—P4—Ag3	53.0 (3)	C75—C70—C71—C72	−0.6 (9)
C45B—P4—C39—C44	−105.8 (7)	P6—C70—C71—C72	−173.3 (5)
C38—P4—C39—C44	147.5 (4)	C70—C71—C72—C73	−0.1 (11)
C45—P4—C39—C44	−107.0 (8)	C71—C72—C73—C74	−0.1 (11)
Ag3—P4—C39—C44	21.5 (4)	C72—C73—C74—C75	0.9 (11)
C45B—P4—C39—C40	75.6 (7)	C71—C70—C75—C74	1.4 (8)
C38—P4—C39—C40	−31.2 (5)	P6—C70—C75—C74	174.7 (5)
C45—P4—C39—C40	74.3 (8)	C73—C74—C75—C70	−1.6 (10)
Ag3—P4—C39—C40	−157.1 (4)	C77—N2—C76—N1	−176.3 (6)
C44—C39—C40—C41	0.2 (8)	C77—N2—C76—S1	4.5 (11)
P4—C39—C40—C41	178.8 (4)	C76—N2—C77—C78	−152.1 (7)
C39—C40—C41—C42	0.5 (8)	C76—N2—C77—C82	32.6 (11)
C40—C41—C42—C43	0.2 (9)	C82—C77—C78—C79	−1.6 (12)
C41—C42—C43—C44	−1.4 (10)	N2—C77—C78—C79	−177.2 (7)
C40—C39—C44—C43	−1.5 (8)	C77—C78—C79—C80	0.1 (14)
P4—C39—C44—C43	179.9 (5)	C78—C79—C80—C81	0.7 (15)
C42—C43—C44—C39	2.1 (9)	C79—C80—C81—C82	0.0 (15)
C39—P4—C45—C50	151.6 (16)	C78—C77—C82—C81	2.2 (12)
C38—P4—C45—C50	−98.6 (18)	N2—C77—C82—C81	177.4 (7)
Ag3—P4—C45—C50	24 (2)	C80—C81—C82—C77	−1.4 (14)

### Hydrogen-bond geometry (Å, º)

**Table:**

*D*—H···*A*	*D*—H	H···*A*	*D*···*A*	*D*—H···*A*
N1—H1*A*···I3	0.86	2.82	3.633 (9)	159
N2—H2*A*···I3	0.86	2.72	3.568 (6)	170
C53—H53···S1^i^	0.99	2.88	3.67 (2)	143
C13—H13*B*···I3	0.99	3.04	3.933 (4)	153

Symmetry code: (i) −*x*+3/2, *y*−1/2, −*z*+3/2.
