# Supplementary figures and images for: Crystal structure of tris­[μ2-bis­(di­phenyl­phosphan­yl)methane-κ2 P:P′]di-μ3-iodido-tris­ilver(I) iodide–N-phenyl­thio­urea (1/1)
Source: Acta Crystallogr E Crystallogr Commun. 2015 Oct 3;71(Pt 11):m187–8. doi: 10.1107/S2056989015017120 (PMC4645055; doi:10.1107/S2056989015017120)

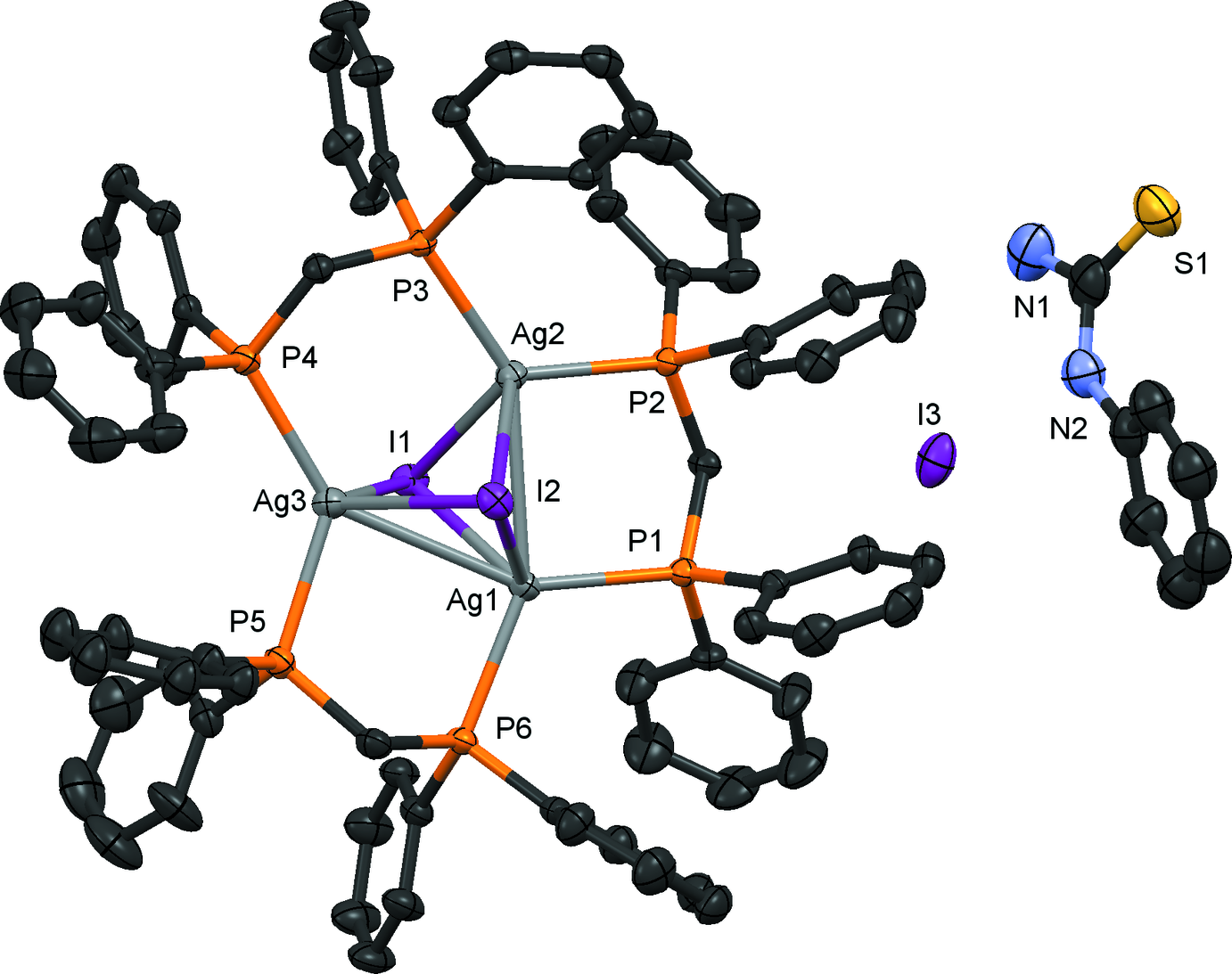

Supplement: Supplementary file 3 [file e-71-0m187-fig1.tif]

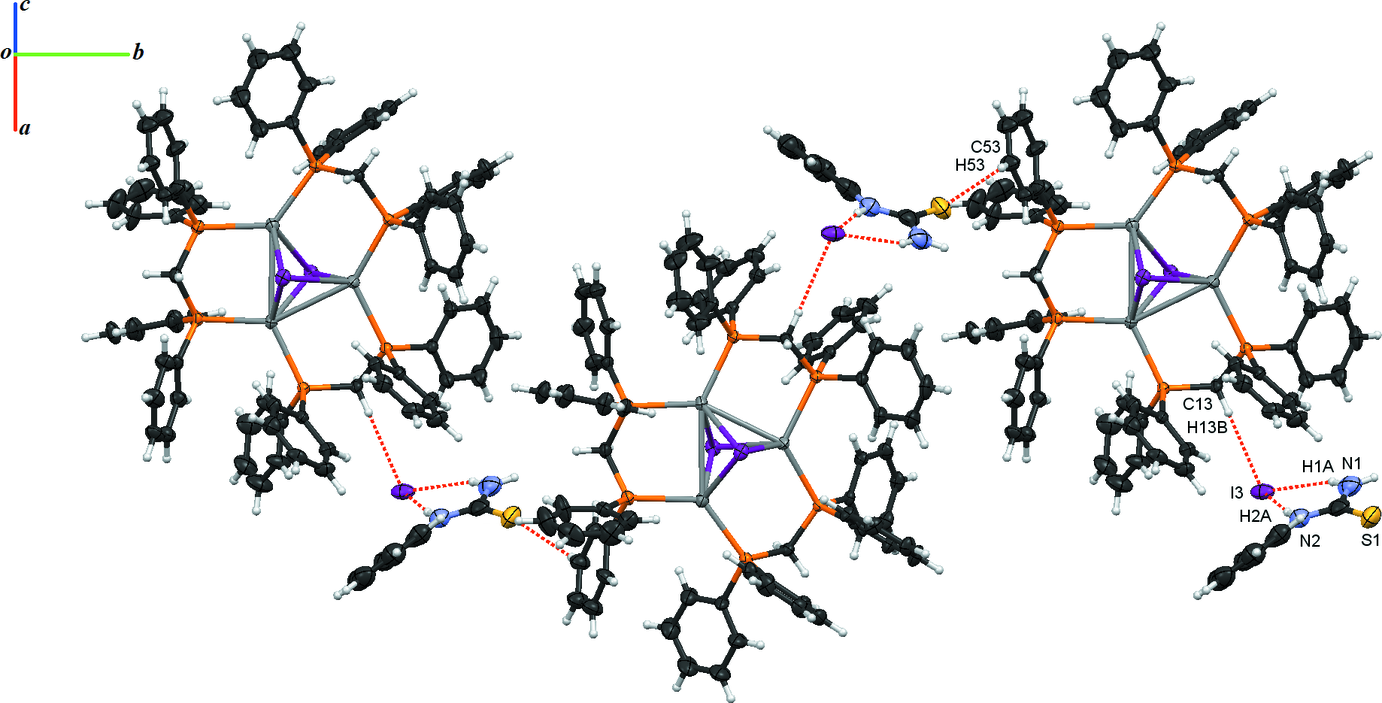

Supplement: Supplementary file 4 [file e-71-0m187-fig2.tif]
